# Machine learning-based multiparametric MRI radiomics for predicting poor responders after neoadjuvant chemoradiotherapy in rectal Cancer patients

**DOI:** 10.1186/s12885-022-09518-z

**Published:** 2022-04-19

**Authors:** Jia Wang, Jingjing Chen, Ruizhi Zhou, Yuanxiang Gao, Jie Li

**Affiliations:** 1 Department of Ultrasound, Qingdao Women and Children Hospital, Shandong Qingdao, China; 2 Department of Radiology, The Affiliated Hospital of Qingdao University, 16 Jiangsu Road, Shandong Qingdao, China

**Keywords:** Rectal cancer, Radiomics, Neoadjuvant chemoradiotherapy, Response, Machine learning

## Abstract

**Background:**

The purpose of this study was to investigate and validate multiparametric magnetic resonance imaging (MRI)-based machine learning classifiers for early identification of poor responders after neoadjuvant chemoradiotherapy (nCRT) in patients with locally advanced rectal cancer (LARC).

**Methods:**

Patients with LARC who underwent nCRT were included in this retrospective study (207 patients). After preprocessing of multiparametric MRI, radiomics features were extracted and four feature selection methods were used to select robust features. The selected features were used to build five machine learning classifiers, and 20 (four feature selection methods × five machine learning classifiers) predictive models for the screening of poor responders were constructed. The predictive models were evaluated according to the area under the curve (AUC), F1 score, accuracy, sensitivity, and specificity.

**Results:**

Eighty percent of all predictive models constructed achieved an AUC of more than 0.70. A predictive model using a support vector machine classifier with the minimum redundancy maximum relevance (mRMR) selection method followed by the least absolute shrinkage and selection operator (LASSO) selection method showed superior prediction performance, with an AUC of 0.923, an F1 score of 88.14%, and accuracy of 91.03%. The predictive performance of the constructed models was not improved by ComBat compensation.

**Conclusions:**

In rectal cancer patients who underwent neoadjuvant chemoradiotherapy, machine learning classifiers with radiomics features extracted from multiparametric MRI were able to accurately discriminate poor responders from good responders. The techniques should provide additional information to guide patient-tailored treatment.

**Supplementary Information:**

The online version contains supplementary material available at 10.1186/s12885-022-09518-z.

## Background

In recent years, preoperative neoadjuvant chemoradiotherapy (nCRT) has gained acceptance as a standard therapy for patients with locally advanced rectal cancer (LARC) [1–3]. Preoperative nCRT is used to achieve tumor shrinkage, and the prognoses of patients with LARC have improved, in part owing to the use of nCRT [4, 5].

Although nCRT plays an important role in improving the prognosis for LARC, the patient responses to nCRT vary from a complete lack of response to pathologic complete response (pCR) [6, 7]. Accurate identification of the tumor response to nCRT is therefore of great value for patients with LARC, allowing ineffective treatment to be avoided. The early identification of poor responders could provide an opportunity for such patients to proceed promptly to extensive surgery, because substantial evidence from previous studies has illustrated that extensive surgery is likely to reduce the local recurrence rate after operation and improve the prognosis for poor responders [8, 9]. Thus, the recent trend for patient-tailored surgery strategies for patients with LARC who have undergone nCRT has highlighted the need for reliable methods to accurately identify poor responders before surgery.

Response prediction in LARC patients who have undergone nCRT generally depends on surgically resected specimens, although this implies a substantial time delay. Previous articles confirmed the potential of medical imaging for evaluating treatment response before surgery, and among the available imaging modalities, magnetic resonance imaging (MRI) is considered to be the most promising method for assessing the response to nCRT in patients with LARC, owing to its good soft tissue resolution and the absence of ionizing radiation [10, 11]. Although a few imaging markers derived from preoperative MRI such as tumor diameter, tumor volume, and apparent diffusion coefficient (ADC) values can offer additional guidance for the screening of poor responders after nCRT [12–18], these imaging markers often achieve only low accuracy in the prediction of poor responders. For ADC values in particular, the results for assessing the response to nCRT are inconsistent [19, 20]. Despite the diverse outcomes of nCRT for LARC, there is still no consensus on which approach is the most reliable for evaluating tumor response after nCRT, and concerns still exist regarding the use of non-invasive methods to assess the response to nCRT earlier in the treatment regime.

Recently, the development of radiomics has shown great potential for evaluating therapeutic effects [21]. By converting medical images into a large number of quantitative features, radiomics can reflect pathophysiology and even tumor heterogeneity [22]. The analysis of radiomics features may provide extra information for tumor classification, as well as for predicting the response to treatment. The potential of radiomics measures as biomarkers has been investigated in rectal carcinoma and a variety of other cancers, including breast cancer [23], cerebral glioma [24], and prostate cancer [25]. Previous research reported that machine learning classifiers using MRI-based radiomics can predict the tumor response to nCRT in patients with LARC [26–29]. However, these previous studies only focused on the prediction of pathological complete response (pCR), and they might have inherent limitations in reflecting the impact of nCRT. As a new approach, the full value of radiomics for the prediction of poor responders needs further investigation.

The aim of this study was therefore to investigate and validate preoperative MRI-based machine learning classifiers for the early identification of poor responders after nCRT in patients with LARC.

## Materials and methods

### Patients

**Fig. 1:**
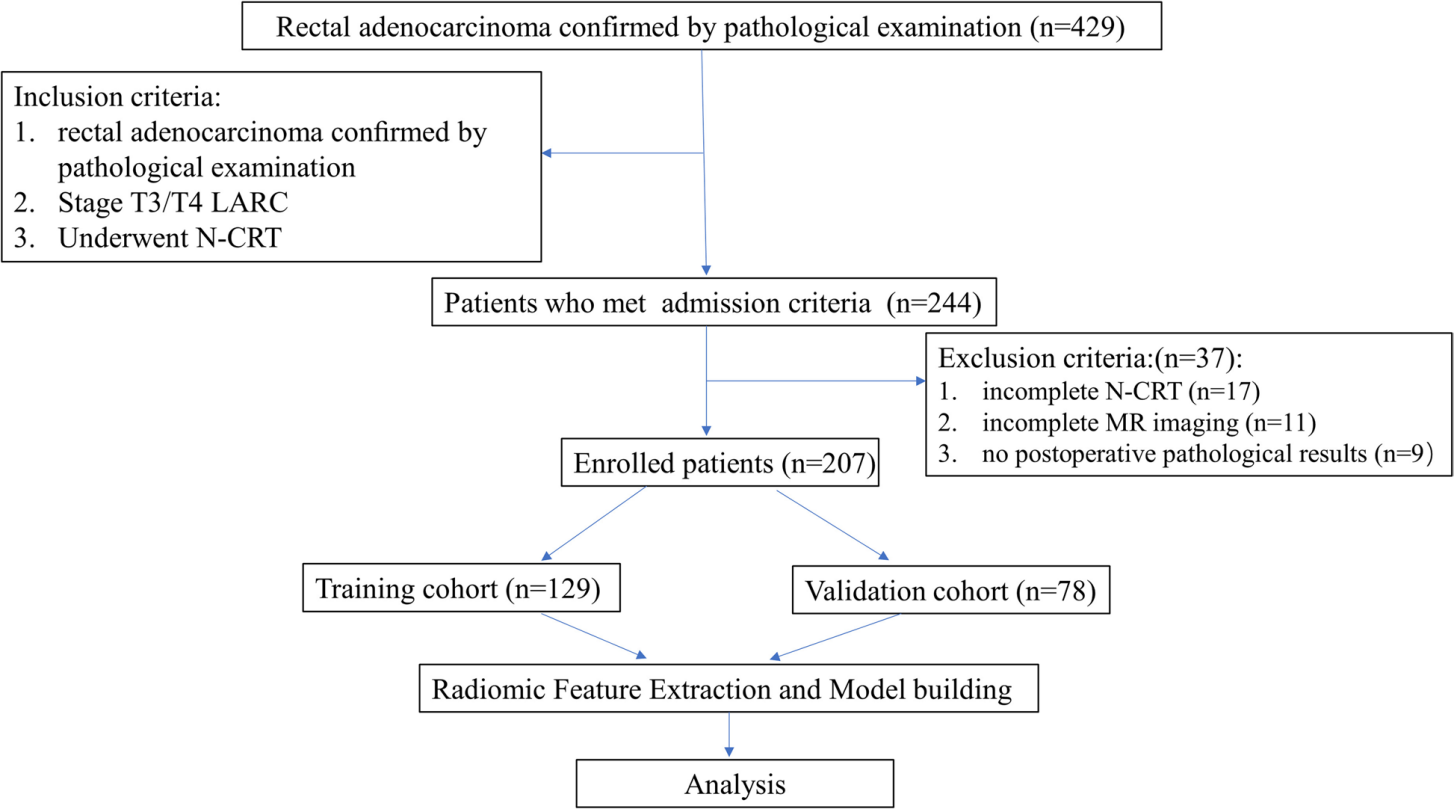
Flow chart of the patient recruitment process

**Fig. 2:**
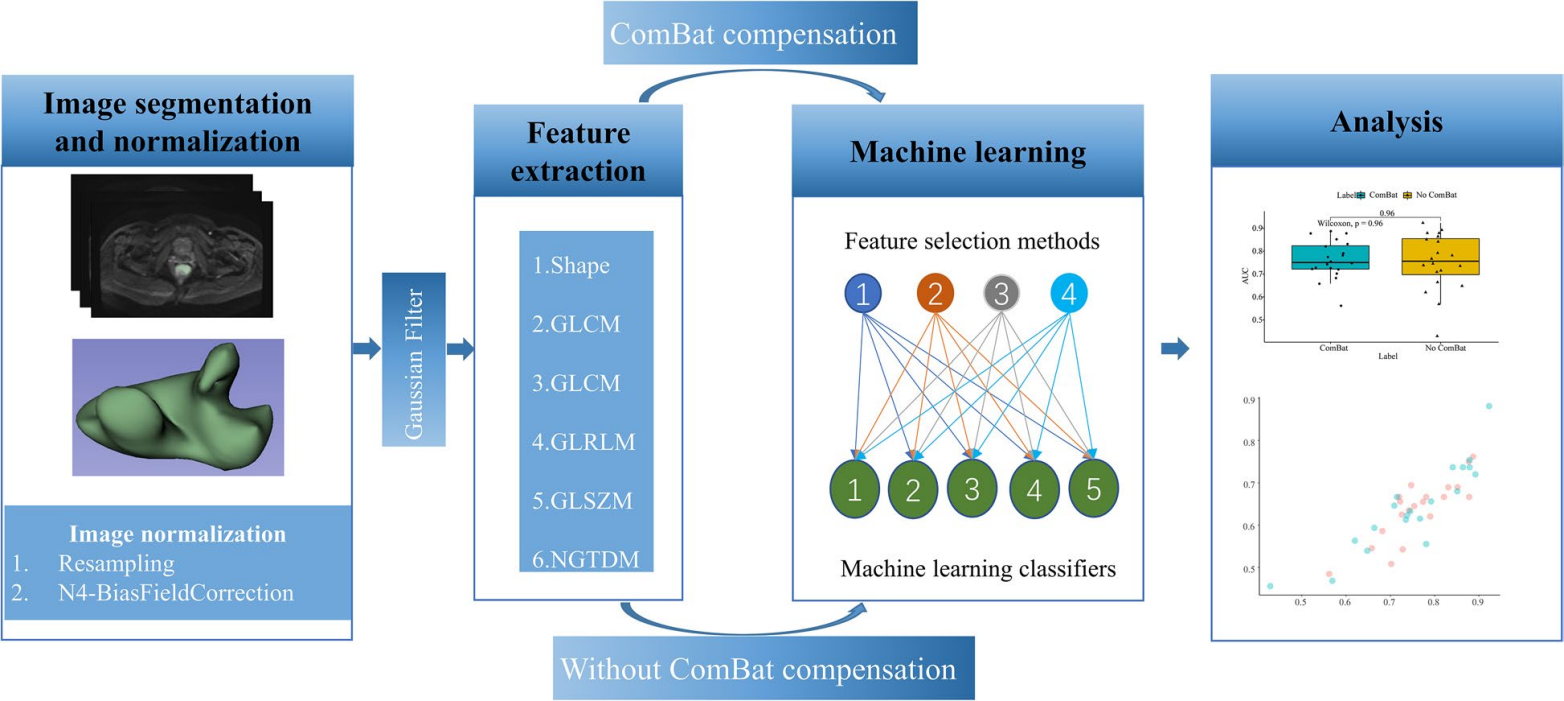
Workflow for the radiomics analysis

Our institutional review board approved this retrospective study and the requirement for written informed consent from patients was waived. Rectal MR images of patients with LARC who received nCRT followed by surgery in our institution between March 2012 and May 2020 were retrospectively analyzed. The inclusion and exclusion criteria and the patient recruitment process are shown in Fig. 1. Patients imaged before Dec 2017 were allocated to a training dataset (*n* = 129), whereas those imaged after this date were allocated to a validation dataset (*n* = 78). Figure 2 illustrates the workflow for the radiomics analysis.

### Pathological assessments of tumor regression

Histopathologic analysis of the surgically resected specimen is considered the gold standard for determining the response to nCRT. Two pathologists who were blinded to the MRI and clinical data evaluated the histopathologic reports, which included the tumor response grading (TRG) according to that proposed by Mandard et al. [30]. The criteria for the TRG are shown in Supplementary Table S1. We classified patients with TRG 1–2 into a good response group, and those with TRG 3–5 into a poor response group.

### MRI acquisition

All patients underwent a magnetic resonance examination on a Signa 3.0-T or Signa HDX 3.0-T MRI scanner (GE Medical Systems, Milwaukee, WI, USA). T2-weighted imaging (T2WI), diffusion-weighted imaging (DWI), and contrast-enhanced T1-weighted imaging (CE-T1WI) were acquired. The MR protocol is outlined in Table 1. Gadolinium contrast (Magnevist; Schering Diagnostics AG, Berlin, Germany) was intravenously administered at a rate of 2.5 ml/s during the contrast-enhanced MRI scan.

**Table 1:** Parameters of the MRI sequences

Scanner	Sequence	b value (s/mm2)	TR (ms)	TE (ms)	image resolution (mm)	Slice Thickness (mm)	Slice Gap (mm)
Signa 3.0-T	T2WI	–	4200	102	0.75× 0.75	3	1
	CE-T1WI	–	680	13	0.6× 0.6	4	0
	DWI	0,800	4000	68	2× 2	6	6
Signa HDX 3.0-T	T2WI	–	3900	90	0.6× 0.6	3	0.5
	CE-T1WI	–	573	20	0.55 × 0.55	3	0
	DWI	0,800	3800	80	1.5 × 1.5	5	5

*T2WI* T2-weighted imaging, *CE-T1WI* contrast-enhanced T1-weighted imaging, *DWI* diffusion-weighted imaging, *TR* relaxation time, *TE* echo time

### Segmentation of tumor imaging and Radiomic feature extraction

First, the N4BiasFieldCorrection algorithm in SimpleITK (Version: 2.0.2, Home-page: http://simpleitk.org/) running in python (Version: 3.7.6) was applied to all of the MR imaging to achieve gray level normalization. Then, an open-source software package (3D-Slicer, version 3.4.2, https://www.slicer.org/) was used for the imaging segmentation, preprocessing, and feature extraction. Volumes of interest (VOIs) were delineated on each slice of the T2WI, DWI, and CE-T1WI. To enhance the differentiation of radiomics features, image preprocessing methods, including resampling to a voxel size of 1 × 1 × 1 mm and Gaussian filtering with sigma set at values of 0.5, 1.0, and 1.5, were applied. The binwidth parameter in 3D-Slicer was set at 25. Finally, radiomics features were extracted from the preoperative imaging data using 3D-Slicer. The radiomics features extracted are listed in Supplementary Table S2.

### Intra-observer and inter-observer agreement

To assess intra- and inter-observer agreement, 62 samples were randomly chosen from the enrolled patients. Radiologist 1, with 11 years of professional experience, performed VOI delineations twice within 1 week for assessment of intra-observer reproducibility. Radiologist 2, with 8 years of professional experience, independently performed VOI delineations once for evaluation of inter-observer reproducibility. First, the Dice coefficient was used to evaluate overlap between the VOIs. The SimpleITK routine running in python was used to calculate the Dice coefficient. Second, all radiomics features extracted from the VOI segmentations performed by the two radiologists were assessed for intra-observer and inter-observer agreement using the intraclass correlation coefficient (ICC). Radiomics features with intra- and inter-observer ICCs ≥0.75 were accepted as having good reproducibility in a previous article [31]; therefore, radiomics features with ICCs ≥0.75 were considered to be robust features.

### The ComBat compensation method

The ComBat compensation method was evaluated for its ability to correct for variations in the radiomic features derived from different MRI scanners (https://github.com/Jfortin1/ComBatHarmonization). The ComBat function was performed using R software (version 3.4.2; R Foundation for Statistical Computing, Vienna, Austria).

### Feature selection methods and machine learning algorithms

Radiomics features extracted from the training cohort were used for the feature selection procedures. Four feature selection methods were included in the study: least absolute shrinkage and selection operator (LASSO), recursive feature elimination (RFE), minimum redundancy maximum relevance feature selection (mRMR), and mRMR combined with LASSO.

Five machine learning algorithms were used to separate poor responders from good responders: decision tree (DT), random forest (RF), support vector machine (SVM), logistic regression (LR), and Adaboost. All feature selection and classification operations were performed using R software.

The combination of four feature selection methods and five machine learning classifiers resulted in 20 (4 × 5 = 20) predictive models. The predictive values of the models were quantified by the receiver operating characteristics (ROC) curve and the area under the curve (AUC), and confusion matrix analysis with indicators of F1 score. Accuracy, sensitivity, and specificity were also used to assess the diagnostic performance of the models. The AUCs of the models were evaluated using the Delong test.

### Statistics

For the clinical data, independent *t*-tests or Mann–Whitney U tests were used to analyze continuous variables. Fisher’s exact test or chi-square tests were used to analyze categorical variables. A two-sided *p* value of < 0.05 was used as the criterion to indicate a statistically significant difference. The R packages used in this study are listed in Supplementary Table S3.

## Results

### Patient characteristics

The patient selection process is displayed in Fig. 1. A total of 207 patients with LARC were enrolled in the study. The clinical characteristics of the patients and their responses to nCRT are presented in Table 2. Patient age and sex showed no significant differences between the two cohorts (*P* > 0.05, both).

**Table 2:** Patients’ clinical characteristics

	Total	*P*	Training cohort (*n* = 129)	Validation cohort (*n* = 78)	*P*
Age (median, [interquartile range])	66, [55-69]	–	66, [56-70]	64, [51-68]	0.103
Sex					
male	132	< 0.001	80	52	0.599
female	75		49	26	
Response to nCRT					
good responders	88	0.031	59	29	0.288
poor responders	119		70	49	

### Analysis of intra-observer and inter-observer agreement

The median Dice coefficient for the intra-observer overlap in VOIs was 0.912, with an inter-quartile range of 0.871–0.953. For inter-observer VOI overlap, the median Dice coefficient was 0.887, with an inter-quartile range of 0.848–0.937.

A total of 1027 radiomics features were extracted from the three MRI modalities of T2WI, DWI, and CE-T1WI. The inter-observer assessment showed a satisfactory agreement rate of 91.82% (mean ICC = 0.862, range 0.012–0.989), while the intra-observer assessment showed a satisfactory agreement rate of 94.27% (mean ICC = 0.901, range 0.030 to 0.996). Finally, 85 radiomics features (ICC < 0.75) were excluded.

### Radiomics features selected with different feature selection methods

The radiomics features selected after application of the four feature selection methods are listed in Supplementary Tables S4, S5, S6 and S7.

### Classifier performance

**Fig. 3:**
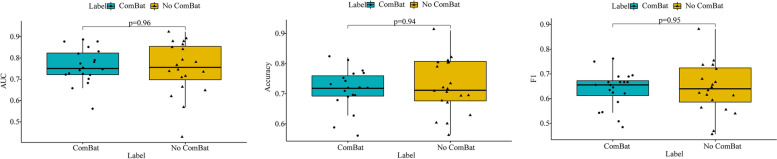
Performance of models with and without ComBat compensation. **a** Comparison of AUC values between ComBat compensation and no ComBat compensation. Comparison of accuracy (**b**) and F1 scores (**c**) between ComBat compensation and no ComBat compensation

No significant difference in AUC, F1 score, or accuracy was observed between classifiers using ComBat compensation and those not using ComBat compensation (Fig. 3), leading us to conclude that the predictive performance of the models was not improved by the use of ComBat compensation.

**Fig. 4:**
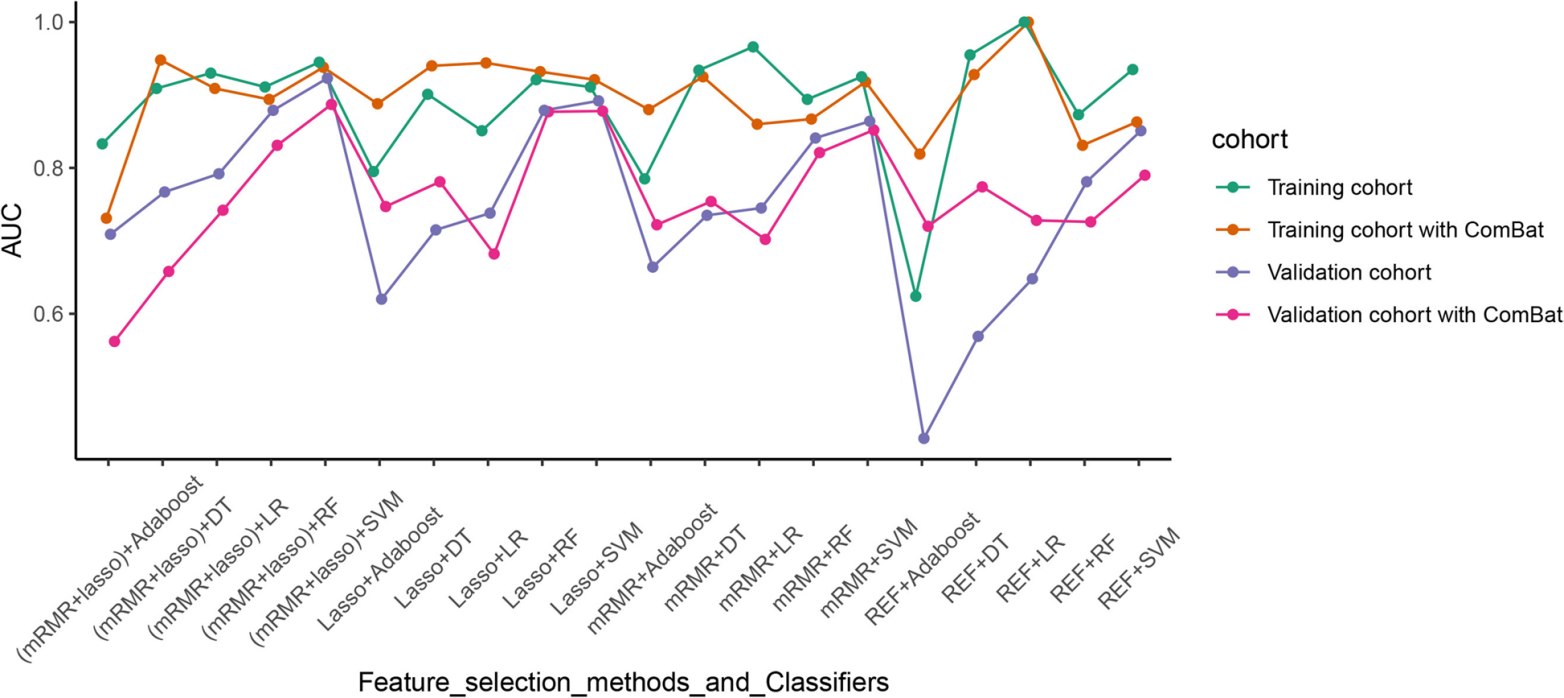
Scatterplots showing the AUCs of classifiers combining different feature selection methods with and without ComBat compensation

The AUCs of the models for the prediction of poor responders in the training cohort and validation cohort are shown in Tables 3 and 4 and Fig. 4. The AUC scores ranged from 0.624 to 1.000 in the training cohort, and from 0.429 to 0.923 in the validation cohort.

**Table 3:** AUCs for the performance of the predictive models in the training cohort

Feature selection methods	Machine Learning Classifiers				
	DT	RF	SVM	LR	Adaboost
No ComBat					
REF	0.955 (0.921-0.989)	0.873 (0.817-0.959)	0.935 (0.896-0.974)	1 (1-1)	0.624 (0.587-0.661)
mRMR	0.934 (0.888-0.98)	0.894 (0.846-0.942)	0.925 (0.864-0.986)	0.966 (0.94-0.992)	0.785 (0.713-0.856)
LASSO	0.901 (0.847-0.956)	0.921 (0.870-0.972)	0.911 (0.863-0.959)	0.851 (0.808-0.894)	0.795 (0.726-0.864)
mRMR+LASSO	0.909 (0.854-0.965)	0.911 (0.862-0.960)	0.945 (0.896-0.994)	0.930 (0.886-0.975)	0.833 (0.767-0.898)
ComBat					
REF	0.928 (0.881-0.975)	0.831 (0.788-0.874)	0.863 (0.812-0.914)	1 (1-1)	0.819 (0.754-0.885)
mRMR	0.925 (0.878-0.973)	0.867 (0.813-1)	0.918 (0.857-0.961)	0.860 (0.827-0.892)	0.88 (0.822-0.937)
LASSO	0.94 (0.899-0.982)	0.932 (0.901-0.963)	0.921 (0.878-0.964)	0.944 (0.908-0.981)	0.888 (0.834-0.941)
mRMR+LASSO	0.948 (0.914-0.982)	0.894 (0.852-0.936)	0.938 (0.895-0.981)	0.909 (0.858-0.96)	0.731 (0.653-0.808)

Values in parentheses are 95% confidence intervals

*LASSO* least absolute shrinkage and selection operator, *RFE* recursive feature elimination, *mRMR* minimum redundancy maximum relevance feature selection, *DT* decision tree, *RF* random forest, *SVM* support vector machine, *LR* logistic regression

**Table 4:** AUCs for the performance of the predictive models in the validation cohort

Feature selection methods	Machine Learning Classifiers				
	DT	RF	SVM	LR	Adaboost
No ComBat					
REF	0.569 (0.441-0.698)	0.781 (0.679-0.884)	0.851 (0.759-0.943)	0.648 (0.534-0.762)	0.429 (0.316-0.542)
mRMR	0.735 (0.622-0.849)	0.841 (0.752-0.931)	0.864 (0.782-0.947)	0.745 (0.628-0.863)	0.664 (0.554-0.775)
LASSO	0.715 (0.597-0.833)	0.879 (0.8-0.957)	0.892 (0.816-0.967)	0.738 (0.627-0.849)	0.62 (0.509-0.731)
mRMR+LASSO	0.767 (0.653-0.882)	0.879 (0.804-0.953)	0.923 (0.862-0.985)	0.792 (0.686-0.897)	0.709 (0.604-0.814)
ComBat					
REF	0.774 (0.669-0.879)	0.726 (0.607-0.844)	0.79 (0.689-0.891)	0.728 (0.608-0.848)	0.72 (0.622-0.818)
mRMR	0.754 (0.648-0.859)	0.821 (0.728-0.915)	0.852 (0.763-0.94)	0.702 (0.578-0.826)	0.722 (0.617-0.827)
LASSO	0.781 (0.675-0.887)	0.877 (0.797-0.958)	0.878 (0.801-0.954)	0.682 (0.572-0.792)	0.747 (0.654-0.841)
mRMR+LASSO	0.658 (0.53-0.786)	0.831 (0.739-0.923)	0.887 (0.806-0.969)	0.742 (0.625-0.86)	0.562 (0.446-0.677)

Values in parentheses are 95% confidence intervals

*LASSO* least absolute shrinkage and selection operator, *RFE* recursive feature elimination, *mRMR* minimum redundancy maximum relevance feature selection, *DT* decision tree, *RF* random forest, *SVM* support vector machine, *LR* logistic regression

**Fig. 5:**
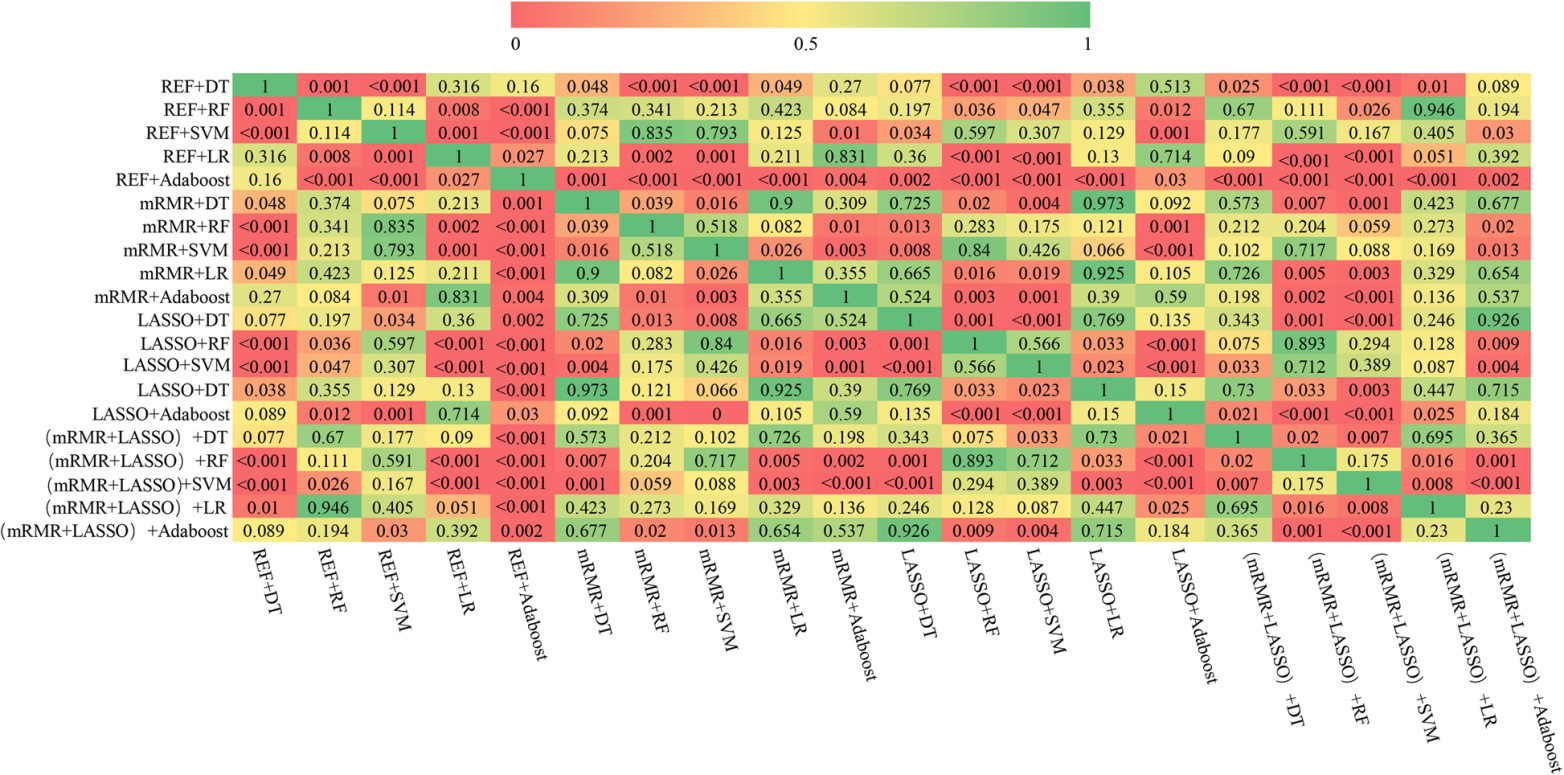
Delong test comparison of the AUCs of the constructed models applied to the validation cohort data. LASSO, least absolute shrinkage and selection operator; RFE, recursive feature elimination; mRMR, minimum redundancy maximum relevance feature selection; DT, decision tree; RF, random forest; SVM, support vector machine; LR, logistic regression

In the validation cohort, the SVM classifier was superior to the other classifiers, with a median AUC of 0.878 (IQR 0.864–0.887). Using the features selected by the mRMR-LASSO analysis, the SVM achieved the highest AUC of 0.923 (CI: 0.862–0.985) in the validation set, followed by LASSO plus SVM (AUC, 0.892). A Delong test comparison of the AUCs of the constructed models for prediction of poor responders in the validation cohort is illustrated in Fig. 5.

**Fig. 6:**
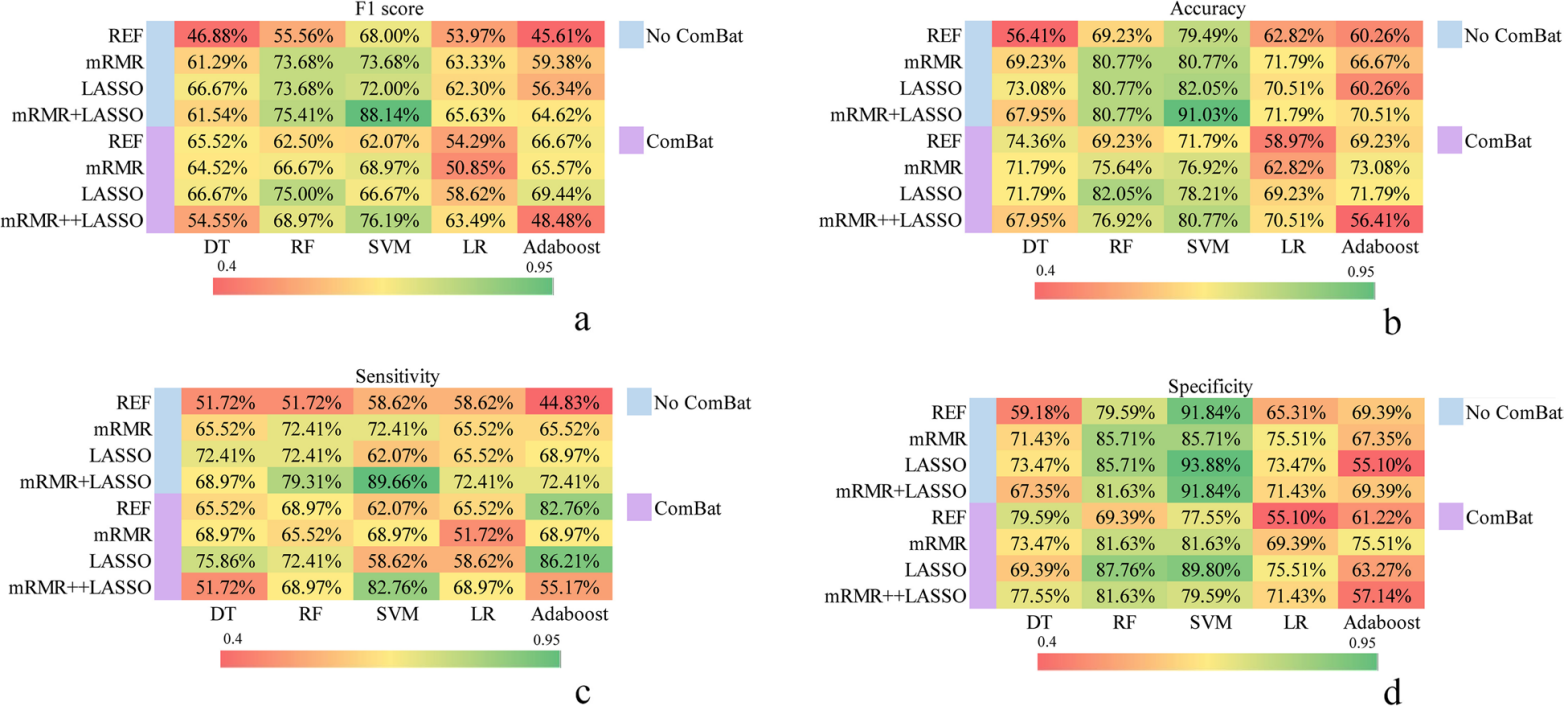
Heatmaps of F1 score (**a**), accuracy (**b**), sensitivity (**c**), and specificity (**d**) for different methods for predicting poor responders

Figure 6 illustrates the accuracy, sensitivity, and specificity values of the constructed models. The model using the mRMR-LASSO feature selection and SVM classifier achieved the highest F1 score (88.14%), accuracy (91.03%), and sensitivity (89.60%). The model using the LASSO feature selection and SVM classifier showed the highest sensitivity at 93.88%, followed by the model using the mRMR-LASSO feature selection and SVM classifier (91.84%).

**Fig. 7:**
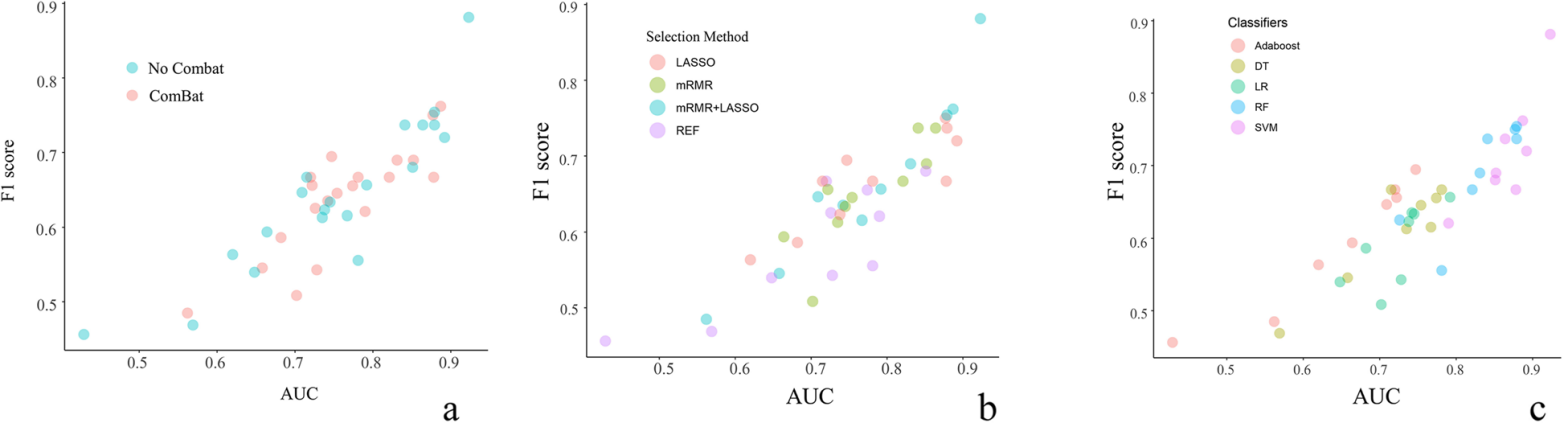
Scatterplot illustrating AUC and F1 scores for different combinations of feature selection methods and machine learning methods applied to the validation set. The circle located in the upper right corner indicates the models with superior prediction performance

Scatterplots of the AUCs and F1 scores (Fig. 7) show that the combination of the mRMR-LASSO feature selection method and SVM machine learning classifier without ComBat compensation achieved the highest performance in the prediction of poor responders.

The radiomics quality scores (QRS) of the current study are listed in Supplementary Table S8.

## Discussion

In the present study, we constructed effective machine-learning classifiers using MRI-based multiparametric radiomics features for the early prediction of poor responders after nCRT. Overall, our results show that 80% of the predictive models achieved AUCs of more than 0.70. We also found that the predictive model using the SVM classifier with the mRMR-LASSO feature selection method showed the highest prediction performance, with an AUC of 0.923, an F1 score of 88.14%, and accuracy of 91.03%. Using this model, patients with a high risk of TRG 3–5 after nCRT could be accurately screened. The method provides a noninvasive and timely approach to screen poor responders before surgery.

From a therapeutic perspective, differentiating poor responders from good responders after nCRT is clinically important for at least two reasons. First, early prediction of poor responders prior to surgery could provide patients with the opportunity to undergo more extensive surgery. According to Denost et al. [8], extensive resection should be adopted in patients who do not respond well to neoadjuvant treatment, to achieve the optimum postoperative and oncological outcomes; patients who did not respond well to nCRT and underwent extensive resection showed 3-year local recurrence significantly lower than those who underwent total mesorectal excision (22% VS. 39%, *p* = 0.04). Second, early prediction of the response to nCRT would allow patients to be divided into different prognostic groups to reduce treatment morbidity associated with second-line chemotherapy drugs and a higher radiation dose.

Pathological evaluation is considered a reliable method to evaluate the response to nCRT and to stratify patients into poor and good responders. However, stratification according to pathological evaluations of surgical specimens involves a substantial time delay for poor responders, and cannot be used to guide tailored surgery strategies. Thus, there is important demand for a non-invasive method to provide accurate prediction of poor responders before surgery.

Previous studies highlighted the value of several imaging modalities for distinguishing poor responders from good responders before surgery. Sun at al. revealed that an increase in mean ADC after nCRT for LARC correlated with a good response to CRT [32]. This correlation between mean ADC and response to nCRT was also found by Lambrecht and colleagues in a study on 20 patients [20]. However, a previous study conducted by Kim et al. included 76 patients with LARC and found that pre-CRT ADC values could not reliably discriminate patients according to their response to nCRT [19]. A possible explanation for these contrasting results is that the mean ADC value derived from a single ROI may underestimate tumor heterogeneity. In another study evaluating the prediction of poor responders after nCRT, Tang and colleagues [33] developed a predictive model using clinical parameters and MRI findings collected using a structured report template to predict poor responders after nCRT, and found an AUC of 0.820. Our best classifier, which used an SVM combined with mRMR-LASSO feature selection, had better predictive performance than their model, with an AUC of 0.923.

The radiomics features used in our study were extracted from multi-parametric MRI, instead of a single imaging modality. These radiomics features extracted from different MRI modalities could reflect tumor heterogeneity caused by variations in tumor intensity, cellularity, and vascularization, and a combination of radiomics features from multi-parametric MRI is likely to improve prognostication in comparison with radiomics features extracted from a single sequence. Although our results do not comprehensively identify the particular MRI sequences that provide the most relevant information for predicting the response to nCRT, radiomics analysis derived from multiparametric MRI clearly has the potential to provide added value to conventional MRI [34, 35]. Nie et al. found that radiomic features derived from T2WI, DWI, and CE-T1WI enhanced the predictive power of an artificial neural network classifier [28]. In another retrospective study including 48 patients, Shi et al. [36] showed that multiparametric MRI features helped to improve the predictive performance of a voxel-based analysis model for identifying pCR after nCRT, a finding in line with that of Liang and colleagues in a cohort of 108 patients [20].

To successfully assess the predictive performance of radiomics-based classifiers, it is necessary to adopt and compare different machine learning classifiers. The application of appropriate machine learning classifiers is likely to help improve the stability and performance of predictive models. We employed AUCs and F1 scores to assess performance, and our results revealed that machine learning using an SVM classifier had the best AUC and F1 score. The superiority of SVM algorithms for building predictive classifiers has been showed in previous studies on other tumor types [37, 38]. SVM classifiers are applicable to the analysis of small datasets and dichotomous variables, and are highly stable.

We also compared the effects of four different feature selection methods reported in previous studies and found that the predictive performance of machine learning classifiers can be affected by the feature selection method. This is consistent with the results of Remeseiro et al. [39]. We found the optimal feature selection method to be mRMR-LASSO, which was also employed in previous studies [40].

In the last phase of the processing, we evaluated the use of ComBat compensation to counteract the effects of different scanners and protocols on the radiomics features while retaining the original definitions of the features. Radiomics feature values can be affected by the use of different scanners [41, 42], and there is a need to lower the variability of radiomics features extracted from different machines [43–45]. However, the predictive performance of our classifiers was not improved by ComBat compensation, which is in accord with a study by Wang et al. [46]. Thus, the value of ComBat compensation for improving the predictive performance of radiomics-based models requires further study.

This study has several limitations. First, this is a retrospective study, and may therefore be subject to selection bias. Some patients on a “wait-and-see” approach were not included in our study. Second, although internal validation of the current study showed optimal AUCs, data with greater variability are required (different scanners, magnetic fields, institutions). Third, because of edema and fibrosis accompanying nCRT, a few tumors showed indistinct margins, which may have impacted on the imaging segmentation. However, MRI demonstrates relatively high tissue contrast compared with other imaging modalities such as computed tomography, making detection of the tumor margin more accurate.

## Conclusion

In this retrospective study, a predictive model based on multiparametric MRI radiomics features and using an SVM classifier and mRMR-LASSO feature selector showed the best performance for presurgical prediction of poor responders to nCRT. The technique should provide additional information to guide patient-tailored treatment plans.

## Supplementary Information

**Additional file 1: Table S1.** The criteria of Mandard standard TRG score is defined as the following. **Table S2.** The types of extracted radiomics features using 3D-Slicer. **Table S3.** The R packages used in this study. **Table S4.** Radiomics features selected by REF. **Table S5.** Radiomics features selected by mRMR. **Table S6.** Radiomics features selected by Lasso. **Table S7.** Radiomics features selected by mRMR combined with LASSO. **Table S8.** The radiomics quality score.

## Data Availability

The datasets used and/or analyzed during the current study are available from the corresponding author on reasonable request.

## Abbreviations

nCRT: Neoadjuvant chemoradiotherapy
LARC: Locally advanced rectal cancer
RFE: Recursive feature elimination
mRMR: Minimum redundancy maximum relevance feature selection
LASSO: Least absolute shrinkage and selection operator
DT: Decision tree
RF: Random forest
SVM: Support vector machine
LR: Logistic regression
IQR: Interquartile range
pCR: Pathological complete response
TRG: Tumor response grading
DWI: Diffusion-weighted imaging
CE-T1WI: Contrast-enhanced T1-weighted imaging
VOI: Volume of interest
CI: Confidence interval
ICC: Intraclass correlation coefficient
RQS: Radiomics quality score
