# Supplementary material for: Machine learning-based multiparametric MRI radiomics for predicting poor responders after neoadjuvant chemoradiotherapy in rectal Cancer patients
Source: BMC Cancer. 2022 Apr 19;22:420. doi: 10.1186/s12885-022-09518-z (PMC9017030; doi:10.1186/s12885-022-09518-z)
Supplement: Supplementary file 1 — Additional file 1: Table S1. The criteria of Mandard standard TRG score is defined as the following. Table S2. The types of extracted radiomics features using 3D-Slicer. Table S3. The R packages used in this study. Table S4. Radiomics features selected by REF. Table S5. Radiomics features selected by mRMR. Table S6. Radiomics features selected by Lasso. Table S7. Radiomics features selected by mRMR combined with LASSO. Table S8. The radiomics quality score. [file 12885_2022_9518_MOESM1_ESM.docx]

**Table S1**

Table S1. The criteria of Mandard standard TRG score is defined as the following:

| pTRG1 | absence of residual tumor tissue on pathological sections and the intestinal wall where the original tumor was located shows fibrotic changes |
| --- | --- |
| pTRG2 | scattered residual tumor cells in fibrotic tissue of the tumors |
| pTRG3 | fibrosis, although there are many residual tumor cells in the tumors |
| pTRG4 | residual tumor cells that significantly exceed the fibrotic range |
| pTRG5 | there is no obvious effect of radiotherapy and chemotherapy |

**Table S2**

Table S2. The types of extracted radiomics features using 3D-Slicer

|  | **Shape Features** |
| --- | --- |
| 1 | VoxelVolume |
| 2 | Maximum3DDiameter |
| 3 | MeshVolume |
| 4 | MajorAxisLength |
| 5 | Sphericity |
| 6 | LeastAxisLength |
| 7 | Elongation |
| 8 | SurfaceVolumeRatio |
| 9 | Maximum2DDiameterSlice |
| 10 | Flatness |
| 11 | SurfaceArea |
| 12 | MinorAxisLength |
| 13 | Maximum2DDiameterColumn |
| 14 | Maximum2DDiameterRow |
|  | **Gray Level Dependence Matrix (GLDM) Features** |
| 1 | GrayLevelVariance |
| 2 | HighGrayLevelEmphasis |
| 3 | DependenceEntropy |
| 4 | DependenceNonUniformity |
| 5 | GrayLevelNonUniformity |
| 6 | SmallDependenceEmphasis |
| 7 | SmallDependenceHighGrayLevelEmphasis |
| 8 | DependenceNonUniformityNormalized |
| 9 | LargeDependenceEmphasis |
| 10 | LargeDependenceLowGrayLevelEmphasis |
| 11 | DependenceVariance |
| 12 | LargeDependenceHighGrayLevelEmphasis |
| 13 | SmallDependenceLowGrayLevelEmphasis |
| 14 | LowGrayLevelEmphasis |
|  | **Gray Level Co-occurrence Matrix (GLCM) Features** |
| 1 | JointAverage |
| 2 | SumAverage |
| 3 | JointEntropy |
| 4 | ClusterShade |
| 5 | MaximumProbability |
| 6 | Idmn |
| 7 | JointEnergy |
| 8 | Contrast |
| 9 | DifferenceEntropy |
| 10 | InverseVariance |
| 11 | DifferenceVariance |
| 12 | Idn |
| 13 | Idm |
| 14 | Correlation |
| 15 | Autocorrelation |
| 16 | SumEntropy |
| 17 | MCC |
| 18 | SumSquares |
| 19 | ClusterProminence |
| 20 | Imc2 |
| 21 | Imc1 |
| 22 | DifferenceAverage |
| 23 | Id |
| 24 | ClusterTendency |
|  | **Gray-level Run-length Matrix (GLRLM) features** |
| 1 | ShortRunLowGrayLevelEmphasis |
| 2 | GrayLevelVariance |
| 3 | LowGrayLevelRunEmphasis |
| 4 | GrayLevelNonUniformityNormalized |
| 5 | RunVariance |
| 6 | GrayLevelNonUniformity |
| 7 | LongRunEmphasis |
| 8 | ShortRunHighGrayLevelEmphasis |
| 9 | RunLengthNonUniformity |
| 10 | ShortRunEmphasis |
| 11 | LongRunHighGrayLevelEmphasis |
| 12 | RunPercentage |
| 13 | LongRunLowGrayLevelEmphasis |
| 14 | RunEntropy |
| 15 | HighGrayLevelRunEmphasis |
| 16 | RunLengthNonUniformityNormalized |
|  | **Gray Level Size Zone Matrix (GLSZM) Features** |
| 1 | GrayLevelVariance |
| 2 | ZoneVariance |
| 3 | GrayLevelNonUniformityNormalized |
| 4 | SizeZoneNonUniformityNormalized |
| 5 | SizeZoneNonUniformity |
| 6 | GrayLevelNonUniformity |
| 7 | LargeAreaEmphasis |
| 8 | SmallAreaHighGrayLevelEmphasis |
| 9 | ZonePercentage |
| 10 | LargeAreaLowGrayLevelEmphasis |
| 11 | LargeAreaHighGrayLevelEmphasis |
| 12 | HighGrayLevelZoneEmphasis |
| 13 | SmallAreaEmphasis |
| 14 | LowGrayLevelZoneEmphasis |
| 15 | ZoneEntropy |
| 16 | SmallAreaLowGrayLevelEmphasis |
|  | **Neighbouring Gray Tone Difference Matrix (NGTDM) Features** |
| 1 | Coarseness |
| 2 | Complexity |
| 3 | Strength |
| 4 | Contrast |
| 5 | Busyness |

**Table S3**

| Table S3. The R packages used in this study | | |
| --- | --- | --- |
| Function | Pakages of R software | Version |
| LASSO | glmnet | 4.0-2 |
| RFE | caret | 6.0-86 |
| mRMR | mRMRe | 2.1.0 |
| Delong’s test | pROC | 1.15.3 |
| ICC | psych | 0.84.1 |
| decision tree | rpart | 4.1-15 |
| random forest | randomForest | 4.6-14 |
| support vector machine | e1071 | 1.7-3 |
| Adaboost | adabag | 4.2 |
| Note：LASSO, least absolute shrinkage and selection operator; RFE, recursive feature elimination; mRMR, Minimum redundancy maximum relevance feature selection | | |

**Table S4**

Table S4. Radiomics features selected by REF

| Radiomics features selected by REF | | Radiomics features selected by REF after ComBat |
| --- | --- | --- |
| 1 | enT1WI.log.sigma.1.5.mm.3D.glrlm.LongRunHighGrayLevelEmphasis | DWI.log.sigma.1.5.mm.3D.glszm.ZoneVariance |
| 2 | DWI.log.sigma.0.5.mm.3D.gldm.DependenceVariance | enT1WI.log.sigma.1.5.mm.3D.glrlm.LongRunHighGrayLevelEmphasis |
| 3 | DWI.log.sigma.0.5.mm.3D.gldm.DependenceNonUniformityNormalized | T2.original.ngtdm.Complexity |
| 4 | DWI.original.glrlm.GrayLevelNonUniformityNormalized | enT1WI.original.glcm.Id |
| 5 | DWI.log.sigma.0.5.mm.3D.glcm.InverseVariance | enT1WI.log.sigma.0.5.mm.3D.glszm.GrayLevelVariance |
| 6 | DWI.log.sigma.1.0.mm.3D.glcm.Idn | enT1WI.log.sigma.1.0.mm.3D.ngtdm.Strength |
| 7 | DWI.log.sigma.0.5.mm.3D.glcm.DifferenceVariance | T2.log.sigma.1.5.mm.3D.glcm.ClusterProminence |
| 8 | DWI.original.gldm.LowGrayLevelEmphasis | T2.log.sigma.1.0.mm.3D.glcm.JointAverage |
| 9 | DWI.original.ngtdm.Coarseness | T2.log.sigma.1.0.mm.3D.glrlm.ShortRunHighGrayLevelEmphasis |
| 10 | DWI.log.sigma.0.5.mm.3D.gldm.HighGrayLevelEmphasis | enT1WI.log.sigma.1.5.mm.3D.glcm.Idm |
| 11 | T2.log.sigma.0.5.mm.3D.glszm.SizeZoneNonUniformityNormalized | DWI.log.sigma.1.0.mm.3D.gldm.DependenceVariance |
| 12 | DWI.log.sigma.1.5.mm.3D.glrlm.RunEntropy | T2.log.sigma.1.0.mm.3D.gldm.LargeDependenceHighGrayLevelEmphasis |
| 13 | DWI.original.glszm.SmallAreaLowGrayLevelEmphasis | enT1WI.log.sigma.1.0.mm.3D.gldm.DependenceVariance |
| 14 | T2.original.glcm.SumEntropy | T2.log.sigma.1.5.mm.3D.ngtdm.Complexity |
| 15 | DWI.original.glcm.ClusterTendency | enT1WI.log.sigma.0.5.mm.3D.gldm.SmallDependenceEmphasis |
| 16 | T2.original.glszm.LowGrayLevelZoneEmphasis | T2.log.sigma.1.0.mm.3D.gldm.SmallDependenceHighGrayLevelEmphasis |
| 17 | DWI.original.glszm.SizeZoneNonUniformityNormalized | T2.original.gldm.LargeDependenceLowGrayLevelEmphasis |
| 18 | DWI.log.sigma.1.0.mm.3D.glrlm.ShortRunEmphasis | DWI.log.sigma.1.0.mm.3D.glcm.Contrast |
| 19 | DWI.log.sigma.1.5.mm.3D.glszm.ZonePercentage | T2.original.shape.Flatness |
| 20 | T2.original.shape.MajorAxisLength | T2.log.sigma.1.0.mm.3D.glszm.LargeAreaEmphasis |
| 21 | DWI.log.sigma.1.0.mm.3D.glcm.ClusterProminence | enT1WI.original.glcm.Autocorrelation |
| 22 | T2.original.shape.LeastAxisLength | DWI.log.sigma.1.0.mm.3D.glcm.SumSquares |
| 23 | T2.original.shape.VoxelVolume | enT1WI.log.sigma.1.0.mm.3D.glcm.MaximumProbability |
| 24 | T2.log.sigma.1.5.mm.3D.glrlm.GrayLevelNonUniformity | DWI.log.sigma.0.5.mm.3D.glcm.DifferenceVariance |
| 25 | T2.original.shape.SurfaceVolumeRatio | enT1WI.log.sigma.1.5.mm.3D.glcm.InverseVariance |
| 26 | T2.original.glcm.Id | DWI.log.sigma.1.5.mm.3D.glrlm.ShortRunEmphasis |
| 27 | T2.original.shape.Maximum3DDiameter | T2.original.glszm.LowGrayLevelZoneEmphasis |
| 28 | T2.original.shape.MeshVolume | enT1WI.log.sigma.1.5.mm.3D.glrlm.GrayLevelNonUniformity |
| 29 | enT1WI.log.sigma.1.5.mm.3D.glrlm.LongRunLowGrayLevelEmphasis | enT1WI.log.sigma.1.5.mm.3D.glcm.ClusterProminence |
| 30 | DWI.log.sigma.1.0.mm.3D.glrlm.GrayLevelNonUniformityNormalized | T2.log.sigma.1.0.mm.3D.gldm.GrayLevelVariance |
| 31 | enT1WI.original.glrlm.RunPercentage |  |
| 32 | T2.original.shape.Flatness |  |
| 33 | enT1WI.log.sigma.0.5.mm.3D.glrlm.LongRunLowGrayLevelEmphasis |  |
| 34 | DWI.log.sigma.1.0.mm.3D.glcm.DifferenceEntropy |  |
| 35 | T2.log.sigma.1.5.mm.3D.ngtdm.Complexity |  |
| 36 | T2.original.glrlm.ShortRunHighGrayLevelEmphasis |  |
| 37 | T2.log.sigma.1.5.mm.3D.glszm.ZoneVariance |  |
| 38 | T2.log.sigma.0.5.mm.3D.glcm.JointEnergy |  |
| 39 | T2.log.sigma.1.5.mm.3D.gldm.SmallDependenceLowGrayLevelEmphasis |  |
| 40 | DWI.log.sigma.1.5.mm.3D.glrlm.LowGrayLevelRunEmphasis |  |
| 41 | DWI.original.glcm.Idm |  |
| 42 | DWI.log.sigma.0.5.mm.3D.glcm.Correlation |  |
| 43 | DWI.log.sigma.1.0.mm.3D.glrlm.HighGrayLevelRunEmphasis |  |
| 44 | T2.log.sigma.1.0.mm.3D.glrlm.GrayLevelNonUniformity |  |
| 45 | DWI.original.gldm.LargeDependenceLowGrayLevelEmphasis |  |
| 46 | enT1WI.log.sigma.0.5.mm.3D.gldm.DependenceNonUniformity |  |
| 47 | enT1WI.log.sigma.1.5.mm.3D.gldm.SmallDependenceEmphasis |  |
| 48 | enT1WI.original.glszm.GrayLevelNonUniformity |  |
| 49 | T2.original.glcm.ClusterShade |  |
| 50 | T2.log.sigma.0.5.mm.3D.gldm.SmallDependenceLowGrayLevelEmphasis |  |

**Table S5**

Table S5. Radiomics features selected by mRMR

| Radiomics features selected by mRMR | | Radiomics features selected by mRMR after ComBat |
| --- | --- | --- |
| 1 | DWI.log.sigma.0.5.mm.3D.ngtdm.Strength | enT1WI.log.sigma.1.0.mm.3D.glcm.Idn |
| 2 | enT1WI.log.sigma.1.0.mm.3D.glcm.Idn | DWI.original.glrlm.HighGrayLevelRunEmphasis |
| 3 | T2.log.sigma.1.0.mm.3D.glszm.LargeAreaEmphasis | T2.log.sigma.1.0.mm.3D.glszm.LargeAreaEmphasis |
| 4 | DWI.original.glrlm.HighGrayLevelRunEmphasis | enT1WI.log.sigma.0.5.mm.3D.glszm.LargeAreaLowGrayLevelEmphasis |
| 5 | T2.log.sigma.1.0.mm.3D.ngtdm.Busyness | DWI.log.sigma.0.5.mm.3D.ngtdm.Strength |
| 6 | DWI.log.sigma.1.5.mm.3D.glcm.Autocorrelation | enT1WI.log.sigma.1.5.mm.3D.glszm.LargeAreaLowGrayLevelEmphasis |
| 7 | DWI.log.sigma.0.5.mm.3D.glrlm.LongRunHighGrayLevelEmphasis | DWI.log.sigma.1.0.mm.3D.glrlm.ShortRunEmphasis |
| 8 | T2.log.sigma.1.5.mm.3D.glszm.ZoneVariance | DWI.log.sigma.1.5.mm.3D.gldm.LowGrayLevelEmphasis |
| 9 | enT1WI.log.sigma.1.5.mm.3D.gldm.LargeDependenceHighGrayLevelEmphasis | enT1WI.original.glszm.HighGrayLevelZoneEmphasis |
| 10 | enT1WI.log.sigma.1.0.mm.3D.gldm.LargeDependenceEmphasis | enT1WI.log.sigma.1.0.mm.3D.glrlm.RunLengthNonUniformity |
| 11 | DWI.log.sigma.1.0.mm.3D.glrlm.ShortRunEmphasis | DWI.log.sigma.1.5.mm.3D.glcm.SumAverage |
| 12 | DWI.log.sigma.1.5.mm.3D.gldm.GrayLevelNonUniformity | DWI.log.sigma.1.0.mm.3D.gldm.HighGrayLevelEmphasis |
| 13 | DWI.log.sigma.1.0.mm.3D.gldm.LargeDependenceHighGrayLevelEmphasis | DWI.log.sigma.1.5.mm.3D.glcm.Autocorrelation |
| 14 | enT1WI.log.sigma.1.5.mm.3D.glrlm.GrayLevelNonUniformityNormalized | DWI.original.glszm.GrayLevelVariance |
| 15 | DWI.log.sigma.1.5.mm.3D.glcm.SumAverage | T2.log.sigma.1.5.mm.3D.glszm.ZoneVariance |
| 16 | DWI.original.glszm.GrayLevelVariance | T2.log.sigma.0.5.mm.3D.glrlm.ShortRunLowGrayLevelEmphasis |
| 17 | DWI.original.glszm.ZoneVariance | DWI.log.sigma.1.0.mm.3D.gldm.LargeDependenceHighGrayLevelEmphasis |
| 18 | enT1WI.log.sigma.0.5.mm.3D.gldm.DependenceNonUniformity | enT1WI.log.sigma.1.0.mm.3D.glrlm.ShortRunHighGrayLevelEmphasis |
| 19 | enT1WI.original.glszm.HighGrayLevelZoneEmphasis | DWI.log.sigma.0.5.mm.3D.glrlm.LongRunHighGrayLevelEmphasis |
| 20 | enT1WI.original.glcm.ClusterTendency | DWI.log.sigma.1.5.mm.3D.glszm.ZoneVariance |
| 21 | DWI.log.sigma.1.0.mm.3D.gldm.DependenceVariance | T2.original.glcm.SumEntropy |
| 22 | enT1WI.log.sigma.1.0.mm.3D.glrlm.RunLengthNonUniformity | enT1WI.log.sigma.1.5.mm.3D.gldm.LargeDependenceHighGrayLevelEmphasis |
| 23 | enT1WI.log.sigma.1.5.mm.3D.glcm.DifferenceEntropy | DWI.log.sigma.1.5.mm.3D.gldm.GrayLevelNonUniformity |
| 24 | T2.original.glcm.SumEntropy | enT1WI.log.sigma.1.5.mm.3D.glrlm.GrayLevelNonUniformityNormalized |
| 25 | T2.original.shape.Flatness | enT1WI.log.sigma.1.5.mm.3D.glszm.HighGrayLevelZoneEmphasis |
| 26 | DWI.original.glszm.LowGrayLevelZoneEmphasis | enT1WI.log.sigma.1.0.mm.3D.gldm.LargeDependenceEmphasis |
| 27 | enT1WI.original.shape.Maximum2DDiameterSlice | DWI.original.glszm.LowGrayLevelZoneEmphasis |
| 28 | enT1WI.original.glszm.SizeZoneNonUniformityNormalized | enT1WI.log.sigma.0.5.mm.3D.gldm.DependenceNonUniformity |
| 29 | DWI.log.sigma.0.5.mm.3D.glszm.LargeAreaLowGrayLevelEmphasis | enT1WI.original.shape.Maximum2DDiameterSlice |
| 30 | DWI.log.sigma.0.5.mm.3D.gldm.HighGrayLevelEmphasis | T2.original.shape.Flatness |
| 31 | enT1WI.log.sigma.1.0.mm.3D.glrlm.ShortRunHighGrayLevelEmphasis | enT1WI.log.sigma.1.0.mm.3D.glcm.DifferenceAverage |
| 32 | enT1WI.log.sigma.1.5.mm.3D.gldm.SmallDependenceEmphasis | enT1WI.original.glszm.SizeZoneNonUniformityNormalized |
| 33 | enT1WI.log.sigma.1.5.mm.3D.glrlm.LongRunHighGrayLevelEmphasis | enT1WI.log.sigma.1.5.mm.3D.glrlm.LongRunHighGrayLevelEmphasis |
| 34 | enT1WI.log.sigma.0.5.mm.3D.glszm.LargeAreaLowGrayLevelEmphasis | DWI.log.sigma.1.0.mm.3D.gldm.DependenceVariance |
| 35 | DWI.log.sigma.0.5.mm.3D.glcm.DifferenceVariance | DWI.log.sigma.0.5.mm.3D.gldm.HighGrayLevelEmphasis |

**Table S6**

Table S6. Radiomics features selected by Lasso

| Radiomics features selected by Lasso | | Radiomics features selected by Lasso after ComBat |
| --- | --- | --- |
| 1 | T2.original.shape.Maximum3DDiameter | T2.original.shape.Maximum3DDiameter |
| 2 | T2.original.shape.Flatness | T2.original.shape.Flatness |
| 3 | T2.log.sigma.0.5.mm.3D.glcm.ClusterShade | T2.original.gldm.SmallDependenceLowGrayLevelEmphasis |
| 4 | T2.log.sigma.1.0.mm.3D.gldm.HighGrayLevelEmphasis | T2.log.sigma.0.5.mm.3D.gldm.DependenceNonUniformity |
| 5 | T2.log.sigma.1.0.mm.3D.glcm.JointEnergy | T2.log.sigma.0.5.mm.3D.glcm.Idmn |
| 6 | T2.log.sigma.1.0.mm.3D.glszm.HighGrayLevelZoneEmphasis | T2.log.sigma.1.0.mm.3D.gldm.HighGrayLevelEmphasis |
| 7 | T2.log.sigma.1.5.mm.3D.glrlm.GrayLevelNonUniformity | T2.log.sigma.1.0.mm.3D.glcm.JointEnergy |
| 8 | T2.log.sigma.1.5.mm.3D.ngtdm.Busyness | T2.log.sigma.1.0.mm.3D.glszm.HighGrayLevelZoneEmphasis |
| 9 | enT1WI.original.shape.Maximum2DDiameterSlice | T2.log.sigma.1.5.mm.3D.ngtdm.Busyness |
| 10 | enT1WI.original.glcm.ClusterTendency | enT1WI.original.shape.Maximum2DDiameterSlice |
| 11 | enT1WI.log.sigma.0.5.mm.3D.gldm.HighGrayLevelEmphasis | enT1WI.original.glcm.ClusterTendency |
| 12 | enT1WI.log.sigma.0.5.mm.3D.gldm.SmallDependenceLowGrayLevelEmphasis | enT1WI.original.glszm.SizeZoneNonUniformityNormalized |
| 13 | enT1WI.log.sigma.0.5.mm.3D.glcm.Autocorrelation | enT1WI.log.sigma.0.5.mm.3D.gldm.SmallDependenceLowGrayLevelEmphasis |
| 14 | enT1WI.log.sigma.0.5.mm.3D.glrlm.GrayLevelNonUniformityNormalized | enT1WI.log.sigma.1.0.mm.3D.glcm.DifferenceAverage |
| 15 | enT1WI.log.sigma.0.5.mm.3D.glszm.LargeAreaLowGrayLevelEmphasis | enT1WI.log.sigma.1.0.mm.3D.glrlm.ShortRunHighGrayLevelEmphasis |
| 16 | enT1WI.log.sigma.1.0.mm.3D.glcm.Imc2 | enT1WI.log.sigma.1.0.mm.3D.glrlm.RunLengthNonUniformity |
| 17 | enT1WI.log.sigma.1.0.mm.3D.glrlm.ShortRunHighGrayLevelEmphasis | enT1WI.log.sigma.1.5.mm.3D.glrlm.GrayLevelNonUniformityNormalized |
| 18 | enT1WI.log.sigma.1.0.mm.3D.glrlm.RunLengthNonUniformity | enT1WI.log.sigma.1.5.mm.3D.glrlm.LongRunHighGrayLevelEmphasis |
| 19 | enT1WI.log.sigma.1.5.mm.3D.gldm.SmallDependenceEmphasis | enT1WI.log.sigma.1.5.mm.3D.glrlm.LongRunLowGrayLevelEmphasis |
| 20 | enT1WI.log.sigma.1.5.mm.3D.glrlm.GrayLevelVariance | DWI.original.gldm.LargeDependenceHighGrayLevelEmphasis |
| 21 | enT1WI.log.sigma.1.5.mm.3D.glrlm.LongRunHighGrayLevelEmphasis | DWI.original.glrlm.LowGrayLevelRunEmphasis |
| 22 | enT1WI.log.sigma.1.5.mm.3D.glrlm.LongRunLowGrayLevelEmphasis | DWI.original.glrlm.ShortRunEmphasis |
| 23 | DWI.original.gldm.LargeDependenceHighGrayLevelEmphasis | DWI.original.glrlm.HighGrayLevelRunEmphasis |
| 24 | DWI.original.glcm.Idmn | DWI.original.glszm.GrayLevelNonUniformity |
| 25 | DWI.original.glrlm.LowGrayLevelRunEmphasis | DWI.original.glszm.ZonePercentage |
| 26 | DWI.original.glrlm.HighGrayLevelRunEmphasis | DWI.original.glszm.SmallAreaEmphasis |
| 27 | DWI.original.glszm.GrayLevelNonUniformity | DWI.original.glszm.LowGrayLevelZoneEmphasis |
| 28 | DWI.original.glszm.LowGrayLevelZoneEmphasis | DWI.log.sigma.0.5.mm.3D.gldm.HighGrayLevelEmphasis |
| 29 | DWI.original.ngtdm.Coarseness | DWI.log.sigma.0.5.mm.3D.glrlm.LongRunHighGrayLevelEmphasis |
| 30 | DWI.log.sigma.0.5.mm.3D.gldm.HighGrayLevelEmphasis | DWI.log.sigma.1.0.mm.3D.gldm.DependenceVariance |
| 31 | DWI.log.sigma.0.5.mm.3D.glcm.MCC | DWI.log.sigma.1.0.mm.3D.gldm.LargeDependenceHighGrayLevelEmphasis |
| 32 | DWI.log.sigma.0.5.mm.3D.glrlm.GrayLevelNonUniformityNormalized | DWI.log.sigma.1.0.mm.3D.glcm.Imc2 |
| 33 | DWI.log.sigma.0.5.mm.3D.glszm.LargeAreaLowGrayLevelEmphasis | DWI.log.sigma.1.0.mm.3D.glrlm.ShortRunLowGrayLevelEmphasis |
| 34 | DWI.log.sigma.1.0.mm.3D.gldm.DependenceVariance | DWI.log.sigma.1.5.mm.3D.glcm.Autocorrelation |
| 35 | DWI.log.sigma.1.0.mm.3D.glrlm.ShortRunLowGrayLevelEmphasis | DWI.log.sigma.1.5.mm.3D.glszm.ZoneVariance |
| 36 | DWI.log.sigma.1.0.mm.3D.glrlm.GrayLevelNonUniformityNormalized |  |
| 37 | DWI.log.sigma.1.5.mm.3D.glszm.ZonePercentage |  |
| 38 | DWI.log.sigma.1.5.mm.3D.glszm.SmallAreaLowGrayLevelEmphasis |  |

**Table S7**

Table S7. Radiomics features selected by mRMR combined with LASSO

| Radiomics features selected by mRMR combined with LASSO | | Radiomics features selected by mRMR combined with LASSO after ComBat |
| --- | --- | --- |
| 1 | DWI.log.sigma.1.0.mm.3D.gldm.DependenceVariance | T2.original.glcm.SumEntropy |
| 2 | enT1WI.log.sigma.1.0.mm.3D.glrlm.RunLengthNonUniformity | enT1WI.log.sigma.1.5.mm.3D.gldm.LargeDependenceHighGrayLevelEmphasis |
| 3 | T2.original.glcm.SumEntropy | DWI.log.sigma.1.5.mm.3D.gldm.GrayLevelNonUniformity |
| 4 | T2.original.shape.Flatness | enT1WI.log.sigma.1.5.mm.3D.glrlm.GrayLevelNonUniformityNormalized |
| 5 | DWI.original.glszm.LowGrayLevelZoneEmphasis | enT1WI.log.sigma.1.5.mm.3D.glszm.HighGrayLevelZoneEmphasis |
| 6 | enT1WI.original.shape.Maximum2DDiameterSlice | enT1WI.log.sigma.1.0.mm.3D.gldm.LargeDependenceEmphasis |
| 7 | enT1WI.original.glszm.SizeZoneNonUniformityNormalized | DWI.original.glszm.LowGrayLevelZoneEmphasis |
| 8 | DWI.log.sigma.0.5.mm.3D.glszm.LargeAreaLowGrayLevelEmphasis | enT1WI.log.sigma.0.5.mm.3D.gldm.DependenceNonUniformity |
| 9 | DWI.log.sigma.0.5.mm.3D.gldm.HighGrayLevelEmphasis | enT1WI.original.shape.Maximum2DDiameterSlice |
| 10 | enT1WI.log.sigma.1.0.mm.3D.glrlm.ShortRunHighGrayLevelEmphasis | T2.original.shape.Flatness |
| 11 | enT1WI.log.sigma.1.5.mm.3D.gldm.SmallDependenceEmphasis | enT1WI.log.sigma.1.0.mm.3D.glcm.DifferenceAverage |
| 12 | enT1WI.log.sigma.1.5.mm.3D.glrlm.LongRunHighGrayLevelEmphasis | enT1WI.original.glszm.SizeZoneNonUniformityNormalized |
| 13 | enT1WI.log.sigma.0.5.mm.3D.glszm.LargeAreaLowGrayLevelEmphasis | enT1WI.log.sigma.1.5.mm.3D.glrlm.LongRunHighGrayLevelEmphasis |
| 14 | DWI.log.sigma.0.5.mm.3D.glcm.DifferenceVariance | DWI.log.sigma.1.0.mm.3D.gldm.DependenceVariance |
| 15 |  | DWI.log.sigma.0.5.mm.3D.gldm.HighGrayLevelEmphasis |

**Table S8**

| Table S8. The radiomics quality score |  |
| --- | --- |
| Criteria | Points |
| Image protocol quality | 2 |
| Multiple segmentation | 1 |
| Phantom study | 1 |
| Imaging at multiple time points | 1 |
| Feature reduction or adjustment for multiple testing | 3 |
| Multivariable analysis | 1 |
| Biological correlates | 1 |
| Cut-off analysis | 0 |
| Discrimination statistics | 1 |
| Calibration statistics | 2 |
| Prospective study | 0 |
| Validation | 2 |
| Comparison to ‘gold standard’ | 2 |
| Potential clinical applications | 2 |
| Cost effectiveness analysis | 1 |
| Open science and data | 1 |
